# Supplementary material for: Are carbon emissions trading and green financial instruments synergistic? -Comprehensive quantitative research based on content analysis
Source: PLoS One. 2024 Mar 7;19(3):e0298601. doi: 10.1371/journal.pone.0298601 (PMC10919601; doi:10.1371/journal.pone.0298601)
Supplement: S1 Data — (ZIP) [file pone.0298601.s001.zip › Data/地级市Z_F工作报告.zip!/╜¡╬≈/╙Ñ╠╢╩╨/└┤╫╘╣1⁄2╓┌║┼í╛┬φ┐╦╩2╛▌═°í┐_2018.docx]

各位代表：

现在，我代表市人民政府向大会报告工作，请予审议，并请各位政协委员和列席会议的同志提出意见。

一、2017年工作回顾

刚刚过去的一年，我们在省委、省政府和市委的坚强领导下，深入贯彻习近平新时代中国特色社会主义思想，紧紧围绕省委“创新引领、绿色崛起、担当实干、兴赣富民”工作方针，按照市委“全域一体、改革创新、优化生态、小市大为”总体要求，扎实推进世界铜都、中华道都、智慧新城建设，经济社会保持平稳健康发展，较好完成了市九届人大二次会议确定的目标任务。

——经济运行稳健。预计完成生产总值820亿元、增长8.6%左右，财政总收入127.7亿元、增长9.3%，一般公共预算收入75.2亿元，规模以上工业增加值增长9%，固定资产投资685.7亿元、增长13.5%，社会消费品零售总额 208亿元、增长12.3%。

——发展质量提高。税收收入占财政总收入比重达85.8%，提高7.7个百分点。市属工业增加值占比达67.9%，提高1.7个百分点。新增高新技术企业30家，高新技术产业实现增加值108.35亿元、增长15%。城镇化率达58.95%，提高1.5个百分点。建成高标准农田10.1万亩，粮食总产73万吨，实现“十四连丰”。

——创新活力增强。人才强市战略深入实施，7家院士工作站挂牌成立，成功申报第一批省级服务支持人才创新创业示范基地，新增各类科技创新平台20家，全社会研发投入占GDP比重4%左右，科技进步贡献率57%。大众创业万众创新蓬勃开展，电子商务交易额达560亿元、增长40%，新增省级电商企业38家。

——民生福祉提升。民生支出90.5亿元，增长2%。城镇居民人均可支配收入31678元、增长8.8%，农村居民人均可支配收入14739元、增长8.9%，各项民生实事完成年度任务，群众生活水平进一步改善。

一年来，主要抓了以下工作：

（一）创新驱动，智慧新城初具雏形。新一代宽带无线移动通信网国家科技重大专项成果转移转化试点示范基地落户我市，移动物联网发展实现网络建设、公共服务平台、产品研发和示范应用全国领跑，建成国内首个三张NB-IoT网络全域覆盖的城市，荣获2017年国际智慧城市建设成果奖、中国智慧城市创新奖。与中国信通院、华为、中兴、浪潮等院企签订46项战略合作协议，现有物联网制造企业31家。移动物联网产业园开园运营，建成中国信通院泰尔物联网研究中心、NB-IoT开放实验室等公共服务平台，移动物联网产业联盟聚集了168家物联网龙头企业和科研机构，孵化产品48种。全球第一个万量级单项NB-IoT商用项目落地鹰潭，成为全国首个主城区全部实现“多表合一”智能抄表和一单式缴费的设区市。完成12万块水表、6400盏路灯、1400个停车位等一批NB-IoT规模化应用，移动物联网连接总数超过8万个。智慧党建、智慧市场监管、国土地质灾害、智慧旅游初步建成全省领先的现代管理新模式，全市61%的政府部门实现数据共享。共享单车、银联云闪付、智能公交、智慧加油站、智慧充电桩等惠民服务示范应用相继建成，“互联网+政务服务”框架基本形成，百姓体验感明显增强。

（二）转型升级，世界铜都建设纵深发力。“八位一体”铜产业体系不断健全，编制世界铜都发展规划，实施红旗铜业三期、博远金属等22个铜精深加工项目，贵冶、三川、江南铜业、广信铜业等4家企业获批国家、省级智能制造示范企业，引进中臻铜业、一朵花铜线灯等一批铜精深加工项目，与北京有色金属研究总院、江铜集团签署战略合作协议，新增3家省级铜产业工程技术研究中心，国内唯一一家国家级铜检中心正式挂牌。铜现货贸易额和铜期货交割仓库业务量持续增长，电解铜、铜材产量分别增长15.8%、9%，铜精深加工占比达43%、提高2个百分点，铜产业实现主营业务收入3400亿元、增长9%。新建标准厂房45.7万平方米，形成铜合金材料、贵溪铜及铜加工、水工、眼镜等4个省级重点产业集群。市现代物流园成为全省首个全国示范物流园区。

（三）彰显特色，中华道都知名度加速提升。启动《鹰潭市中华道都建设发展规划（2017—2020）》编制，大上清宫遗址保护性设施建设工程获国家文物局立项批复，《龙虎山道教文献集成》完成大纲编撰，举办第九届海峡两岸道文化交流论坛、首届中华道家养生学术交流会、对话老子书画精品展等大型活动，提升了我市道文化知名度和影响力。旅游“双创”工作稳步推进，“1+3+N”旅游综合体制改革取得重大突破，举办 2017中国（鹰潭）全域旅游高峰论坛、心学之源暨象山书院建院830周年高端论坛、华泉小村国际文化艺术节和金足迹旅游盛典，直升机运营基地开通低空观光游览，旅游“厕所革命”三年行动计划超额完成，花语世界二期基本建成，白鹤湖景区、贵塘沿线乡村旅游、锦江古镇等重点旅游项目加速推进，青茅境景区入选全省首批森林体验基地，上清古镇获批省级旅游风情小镇，灵溪小镇获批省4A级乡村旅游点，鹰潭入选全国森林旅游示范市，被央视《魅力中国城—2017中国旅游盛典》授予“魅力城市”称号。全市接待国内游客、旅游总收入分别增长21.5%、28.7%。

（四）奋力攻坚，发展内生动力不断释放。余江县宅改908个试点自然村已验收752个，退出宅基地28851宗3961亩，退出宅基地复垦693亩，得到了省委、省政府和国土资源部的充分肯定。全域推开农村宅基地管理试点，1016个试点村退出宅基地11301宗1624亩。国有农垦土地使用权确权和发证率达90%，全国农垦现场推进会在我市召开。农村土地承包经营权确权登记颁证工作在全省首个完成全域验收，农村土地承包经营权抵押贷款试点有序推进，农村土地流转面积占家庭承包土地总面积的40%，新增各类新型农业经营主体310家。“三去一降一补”取得新成效，降成本优环境行动累计为企业减负25.6亿元，鹰潭高新区直供电交易改革试点稳步推进，全市商品房总库存量和住宅库存去化时间分别降为17个月、9个月。“双随机一公开”深入推进，市本级行政许可标准化建设基本完成，全国、全省机构编制“双试点”全面启动，市县乡权责清单实现“全覆盖”。筹建了交建投集团、鹰旅集团公司，理顺了白鹤湖示范区、物流、招商工作体制，推进了卫计等综合执法体制改革。设立全域发展引导基金，新增“新三板、四板”挂牌展示企业10家、150家。举办北京、深圳电子信息暨物联网产业推介会等10余场重大招商活动，全市引进省外2000万元以上项目实际进资348亿元、增长10%，实际利用外资2.88亿美元、增长9%。外贸出口58.45亿元、增长9.43%，连续八年被评为中国外贸百强城市。

（五）提升品质，城乡融合发展协调推进。《鹰潭市城乡总体规划（2015-2030）》及“多规合一”编制完成，320国道月湖段拓宽、环城东路延伸、林荫西路综合改造、龙虎山北路延伸、市中心广场人防工程除险加固、环行绿道沿江骑行公园等项目竣工，院里路贯通、一江两岸城市景观亮化提升、信江北路绿化提升等项目春节前建成，余信贵大桥、余信贵大道、小街小巷改造等项目加速推进。深入开展城乡环境综合整治行动，户外广告（店牌店招）、铁路沿线环境、校园周边环境、建筑垃圾清理等专项整治成效明显，城市水体整治有序实施。新农村建设“三年扫一遍”开局顺利，建成了1185个新农村建设村点，重点打造了9条示范线、10个示范片、14个示范点，实现省市级贫困村和宅改村点新农村建设全覆盖。完成通自然村公路400公里、危桥改造25座、道路安保工程100公里。塘湾清溪小镇、中童眼镜小镇列为省级第一批特色小镇。

（六）持续发力，生态文明建设成效显著。国家城乡生活垃圾第三方治理试点全面铺开，建成智能垃圾驿站420个，垃圾焚烧发电厂一期点火投产。实施大气、水、土壤污染防治计划，深入开展绿色生态农业十大行动和“清河行动”，禁养区88家规模畜禽养殖场全部关停拆除，病死畜禽无害化处理中心投入运行，建成贵冶周边重点区域重金属污染治理二期项目，完成人工造林2.74万亩。全市空气质量优良率85%，PM10浓度全省最低，水环境功能区水质达标率100%，主要河流断面Ⅱ—Ⅲ类水质达标率100%，集中式饮用水水源地水质达标率100%，是全省唯一重点断面没有出现劣V类水质的设区市。贵溪成功创建国家园林城市，贵溪国家森林公园入选“中国森林氧吧”

（七）提标扩面，百姓幸福指数明显增强。脱贫攻坚纵深推进，整合财政涉农扶贫资金5.24亿元，在精准识别、精准分类的基础上，建立了“基本医疗保险、大病医疗保险、二次补偿、商业补充保险、民政医疗救助”五道保障线，产业扶贫实现全覆盖，安居扶贫2016年度2413户任务全部完成，退出省级贫困村6个、市级贫困村7个、贫困人口5751人，贫困发生率由年初的1.93%下降到1.29%。城镇新增就业人数2.54万人，新增转移农村劳动力1.55万人。城乡居民基本医疗保险制度整合工作全面完成，实现跨省异地就医住院医疗费用直接结算。城乡低保保障标准提高到每人每月530元、305元。棚户区改造2016年3267户任务全部完成，2017年4183户全部开工，建成保障性住房359套，新推出公共租赁住房1787套。市师范附小新校区、市二中北校区、人民医院信江分院投入使用，高新区义务教育均衡发展通过国家评估认定。医药卫生体制改革稳步推进，城市公立医院改革住院次均费用下降95元，药占比下降3个百分点。市区更新启用70台新能源纯电动公交车。举办市第八届运动会暨第二届体育文化节、全国古典式摔跤青年锦标赛，许哲怡获得我市历史上首枚全运会金牌。举办全国雕刻职业技能竞赛总决赛。我市荣获全国唯一的“中国微诗城”称号。樟坪畲族乡被评为全国民族团结进步创建示范区。程永林同志荣获全国公安系统一级英雄模范。平安法治鹰潭建设扎实推进，妥善解决了房地产等领域历史遗留问题，道路交通秩序、交通安全、治超等整治工作扎实推进，有效处置了“5·15”等交通事故，建立了拖欠农民工工资系统性治理工作机制，安全生产、食品药品安全形势平稳，防汛工作取得全面胜利，社会保持和谐安定，我市再次荣获全省“平安杯”。

一年来，我们坚持以开展“两学一做”学习教育常态化制度化为契机，着力加强政府自身建设。“放管服”改革持续推进，推行“最多跑一次”和“一次不跑”改革，建立重大项目绿色通道，减证便民实现“二十八证合一”，取消和调整市本级行政权力事项82项，行政效能和服务水平进一步提高。法治政府建设不断加强，办理人大代表建议140件、政协提案248件，满意率均为100%。认真履行全面从严治党主体责任和“一岗双责”，严格落实中央八项规定精神和国务院“约法三章”，政府廉政建设不断加强。

各位代表，一年来的成绩和进步来之不易。这是省委、省政府和市委坚强领导、科学决策的结果，是全市人民攻坚克难、砥砺奋进的结果，是社会各界关心支持、鼎力相助的结果。在此，我代表市人民政府，向在各个岗位上贡献智慧、付出心血和汗水的全市人民，向各民主党派、工商联、无党派和社会各界人士，向驻鹰单位、人民解放军、武警部队官兵和离退休老同志，表示衷心的感谢，并致以崇高的敬意！

需要向代表说明的是，受经济下行压力等因素影响，固定资产投资、社会消费品零售总额、农村居民人均可支配收入等指标略低于预期目标，市妇幼保健新院等项目滞后时序进度。同时，我们也清醒地看到，经济社会发展还存在不少困难和问题：发展不平衡不充分问题仍较突出，经济总量仍然偏小，科技创新能力不强，产业转型任重道远，新旧动能转换还需更大努力。城市功能设施欠账较多，中心城区能级不高、辐射带动能力不强，城乡融合发展水平有待提升。农村、民生等领域短板较为明显，教育、医疗等公共服务与群众期待还有差距，城乡居民持续增收渠道不多。转变政府职能、干部运用新理念推动发展的能力有待增强，少数部门和工作人员不作为、不担当等问题仍然存在。对此，我们将采取有力措施，切实加以解决。

二、2018年主要工作

政府工作报告

2018年是贯彻党的十九大精神的开局之年，是改革开放40周年，也是决胜全面建成小康社会、实施“十三五”规划承上启下的关键一年，做好政府各项工作至关重要。今年政府工作的总体要求是：全面贯彻落实党的十九大精神，以习近平新时代中国特色社会主义思想为指导，按照市委“全域一体、改革创新、优化生态、小市大为”总体要求，坚持稳中求进总基调，以新发展理念为引领，以推动高质量发展为要求，以供给侧结构性改革为主线，以建设“两都一城”为统揽，统筹推进稳增长、促改革、调结构、优生态、惠民生、防风险各项工作，不忘初心、牢记使命，努力开创决胜全面小康、建设富裕美丽幸福鹰潭新局面，奋力谱写新时代中国特色社会主义发展的鹰潭篇章。

今年全市经济社会发展的主要预期目标是：生产总值增长8.5%左右，财政总收入增长8%，一般公共预算收入增长6.5%，规模以上工业增加值增长8.6%左右，固定资产投资增长12%，社会消费品零售总额增长12%，城镇居民人均可支配收入增长8.5%，农村居民人均可支配收入增长8.6%，居民消费价格总水平涨幅控制在3%以内，完成省下达的节能减排计划任务。

围绕实现上述目标，重点实施“七大行动计划”：

（一）实施创新驱动战略行动计划，着力汇聚“两都一城”建设新动能。紧紧抓住新经济时代“互联网正成为基础设施，大数据正成为生产资料，云计算正成为公共服务”的战略机遇，始终把创新摆在全局发展的核心位置，以移动物联网为引领，以推进科技创新为核心，以推动人才强市为支撑，努力建设全国一流智慧新城。

基本建成智慧新城。围绕“三步走”目标，实施移动物联网网络提升工程，持续完善NB-IoT网络覆盖和连接质量，加快eMTC网络商用部署，实现NB-IoT与eMTC融合发展。实施移动物联网平台支撑工程，推进智慧新城云中心、决策指挥中心、体验中心和移动物联网产业园建设，深化移动物联网产业联盟合作，形成集研发制造、系统集成、示范应用于一体的公共服务体系。实施移动物联网产业“万千百”工程，引进一批芯片、模组、平台和终端应用企业，推动一批移动物联网产品进入商用推广，开展智能水表、智能装备等标准制定，力争全市物联网企业达70家以上、产值突破200亿元。实施移动物联网集成应用工程，加快推进“互联网+政务服务”、金融便民服务、智慧社区、智慧能源等项目建设，基本建成城市管理、环境保护、综合治理等政府管理支撑体系和教育、就业、健康等便民服务平台，让智慧新城建设成果惠及全体市民，力争列入国家新型智慧城市试点。

着力推进科技创新。实施创新驱动“5511”工程和科技创新“四个一”工程计划，推进新一代宽带无线移动通信网国家科技重大专项成果转移转化试点示范基地建设，新组建10个省级以上科技创新平台，新增15家以上高新技术企业、30家以上科技型中小企业，实现高新技术产业增加值增长12%以上。健全产学研用创新体系，支持企业设立院士、博士后科研工作站和实践基地，完善科技成果转化服务链。加大规模以上企业研发投入和以科贷通为重点的社会研发投入，推进科技金融试点示范和知识产权质押贷款工作，力争全社会研发投入占GDP比重保持4%左右。推进大众创业、万众创新，鼓励技术创新、模式创新、业态创新，支持鹰潭高新区争创国家自主创新示范区、省级“双创”示范基地。

全力建设人才强市。深入实施“鹰才计划”“乡贤回归”行动计划，推进高层次人才挂职锻炼工作，培养引进一批科技领军人才、实用型高技能人才、企业经营管理人才和创新团队。深化人才体制机制改革，每年设立1000万元人才发展专项资金，赋予省级服务支持人才创新创业示范基地更加灵活宽松的改革权和试验权。加速人才工作信息化建设，建立健全市级人才信息库、鹰籍在外乡贤库、网上综合服务平台等，加速构建竞争力较强的区域性人才小高地。

（二）实施产业转型升级行动计划，着力厚植“两都一城”建设新支撑。按照争当全省产业转型升级先行者的要求，牢牢把握信息化、智能化、融合化、绿色化发展方向，加速发展新经济新业态，大力改造提升传统产业，实现新旧动能协同发力，加快构建现代化经济体系。

培育新制造经济。全力创建“中国制造2025”试点示范城市，开展技术改造深化年活动，力争全年工业技改投资占工业投资比重15%以上。积极创建国家新型工业化产业示范基地，开工建设欧菲科技物联网模组、沃得尔汽车传感器等项目，推动渥泰环保智能净水器、永威光电智能安防监控镜头等项目扩产增效，力争省级以上智能制造试点示范企业达7家以上，数字化车间达50个以上。完善“八位一体”铜产业体系，全面深化与江铜集团合作，积极推动铜检中心引入战略合作伙伴，加快全国铜及铜合金循环利用产业知名品牌创建试点区建设，推进胜华金属二期、贵义铜业、龙轩铜业等项目达产，推动产业链向电子信息和电力电气领域延伸，力争铜精深加工产品占比达45%。加速发展绿色水工、光电新能源、大健康、眼镜等优势产业，力争非铜产业主营业务收入达390亿元、增长10%。推动开发区改革和创新发展，强化开发区的经济管理职能，积极推行开发区政企分开、政资分开。加快建设智能终端产业园、电线电缆产业园、电子信息产业园，完成白露科技园3.62平方公里、五湖健康产业园1平方公里拓园工作，建成标准厂房40万平方米以上。支持贵溪经开区创建国家级经济技术开发区。

发展新服务经济。以打造中华道都为目标，加快推进正一观二期改造，筹办龙虎山道文化国际旅游节，建成道文化主题雕塑公园，力争大上清宫遗址成功申报全国考古十大新发现，推动道文化显性化、大众化、体验化。以旅游“双创”工作为主线，围绕“红魂、绿魄、古韵、墨香”四篇文章，启动天门山旅游综合开发，推进上清古镇改造提升等重点旅游项目建设，大力培育健康养生、乡村旅游、低空观光旅游、精品民宿等新业态，力争成功创建国家全域旅游示范区，全市旅游接待人次、旅游总收入分别增长25%、30%以上。以发展新服务业态为方向，推进中国邮政鹰潭邮件处理及物流仓储中心、林安智慧物流园区等项目，搭建全市物流公共信息平台，加快“互联网+流通”建设，积极培育文娱、家政等消费增长点，形成更多新商业模式。

振兴实体经济。继续开展降成本优环境专项行动，强化挂点帮扶制度，有效降低企业要素成本和制度性交易成本，力争全年新增入规企业25家。稳妥推进售电侧改革，推动重点园区、重点企业参与直供电交易。用好担保平台、财园信贷通等政策，开展铜供应链融资和应急转贷业务，做实小微企业金融服务，提升实体经济贷款占比。积极引导实体店发展，加快线上线下融合发展。弘扬企业家精神，厚植工匠文化，引导企业突出主业、做精产品、做优服务，建设质量强市，推动鹰潭产品向鹰潭品牌转变。

（三）实施城市双修提升行动计划，着力增添“两都一城”建设新魅力。以“城市双修”为抓手，推进城市有机更新、转型发展，完善城市功能，改善人居环境，彰显特色文化，把城市建设得更加宜居宜业，让广大市民共享更有品质的生活。

坚持规划引领。强化空间管控，利用“多规合一”成果，完成空间规划编制，划定“三区”和“三线”。完善城市设计，控制城市轮廓、重要视线、特色地段、关键节点，明确建筑特色、公共空间和景观风貌等管控要求，合理规划城市水系布局，形成“一江两岸”城市设计最终成果。完成停车设施、街道设计、城市色彩控制等专项规划编制和城市排水（雨污）防涝规划编制，推进城市建设从扩容向提质转变。

推进城市修补。完善交通体系，建成余信贵大桥、鹰东鹰南高速收费站、鹰南公交枢纽站和公交智能调度中心等项目，实施11条城区主次干道改造，推进余信贵大道、鹰潭大桥拆旧建新等城市贯通工程，实施206国道余江段改线和龙虎山段改造、320国道余江段改造、万年至鹰潭高铁北站一级公路改建、上清至冷水道路改造等城际畅通工程。提升功能配套，推进高铁商务区、信江新区中央生态公园、应用工程学校搬迁、妇幼保健新院等新区建设项目，完成45条小街小巷改造和杏园、军民路菜场新改建，新改建主城区公厕33座、景区公厕26座。加大棚改力度，加快二二三地质队片区、防腐厂片区、东湖片区、西门道口等棚户区改造和白马李家、朱埠杨家夏家刘家等城中村改造工作，实施信江新区麒麟大道沿线流洪祝家虎岭等村庄房屋征收工作。强化精细管理，积极创建省“公交城市”，更新新能源公交车33辆，新辟和优化公交线路4条。持续开展道路运输市场秩序、违章搭建、占道经营、户外广告、“牛皮癣”等整治活动，塑造精致、精美的良好城市形象。

加强生态修复。全面融入全省国家生态文明试验区建设，推进生态文明试验区“六大体系”建设，让鹰潭蓝天常驻、青山常在、绿水长流。按照居民出行“300米见绿、500米入园”要求，建设一批漫游绿道、街边小游园小花园，新增市区绿地1万平方米。加大重要生态保护区、水源涵养区、江河源头区的生态修复和保护力度，推进东湖、西外湖、东川河综合整治及白露河景观提升等项目，实施19条14公里城市主次干道雨污分流管网改造提升，已建成污水处理厂全部投入运行。实施森林质量提升工程，确保森林覆盖率稳定在58.2%以上。推进园区循环化改造、城市矿产、资源综合利用等试点工程，开展绿色生态农业十大行动，发展装配式建筑、低碳交通，推进公共机构节能，倡导全民绿色低碳、文明健康的生活方式。做好自然资源确权登记工作，全面开展党政领导干部自然资源离任审计，建立健全生态环境保护长效机制。

（四）实施乡村振兴战略行动计划，着力夯实“两都一城”建设新基础。按照产业兴旺、生态宜居、乡风文明、治理有效、生活富裕的总要求，推进农业供给侧结构性改革，健全城乡融合发展体制机制，加快农业农村现代化步伐，努力让农业强起来、农村美起来、农民富起来。

构建现代农业体系。围绕建设“一田”，加强耕地保护，力争新增耕地率3%以上，新建高标准农田4万亩，产业结构调整达30%以上，粮食种植面积和产量稳定在178万亩、70万吨以上，确保粮食安全。围绕打造“三线”，以白鹤湖大道、贵塘公路、320国道杨溪段沿线为主战场，大力实施农业结构调整行动计划“1+9”方案和林下经济发展行动计划“1+6”方案，积极发展休闲农业、乡村旅游、农产品加工等新业态，在一二三产融合发展上培育新增长点。围绕做强“四园”，重点建设白鹤湖、雷溪、杨溪、锦江等4个现代农业示范园区，完善园区基础设施，促进农业龙头企业、新型农业主体、先进农业生产要素向园区集聚。坚持质量兴农、绿色兴农，开展特色农产品标准化生产示范，新增“三品一标”认证产品20个。实施“互联网+现代农业”行动，完善农村电商服务体系，形成线上线下融合、农产品进城和农资、消费品下乡的双向流通格局。

深化农业农村综合改革。统筹推进农村土地三项改革试点工作，全面完成余江农村宅改试点，深化农村宅基地管理全域试点工作，固化试点成果，建立长效机制，整体推进“一改促六化”工作。完成房地一体的集体建设用地使用权和宅基地使用权确权登记工作，确保发证率达95%以上。认真落实“保持土地承包关系稳定并长久不变，第二轮土地承包到期后再延长三十年”的政策，通过土地流转、土地股份合作、联合或土地托管等多种方式，发展适度规模经营，新增各类新型农业经营主体300家以上。扶持发展村级集体经济，确保集体资产保值增值。积极推进农村集体产权制度改革，稳步推进国家农村集体股份权能改革试点、农村土地经营权抵押贷款试点。统筹推进林业、水利、供销、农垦和绿色殡葬等改革。

打造美丽乡村升级版。围绕“三年扫一遍”部署，深入实施新农村建设“整洁美丽、和谐宜居”行动，做多示范点、做精示范线、做靓示范片，重点打造“三线三片”，完成 1000个左右新农村村点建设任务。持续开展城乡环境综合整治攻坚行动，深入推进违法建房专项治理，推行城乡生活垃圾分类试点，加快实施市焚烧发电厂二期扩建及飞灰填埋场项目，完善城乡生活垃圾一体化处理体系。支持贵溪创建全国城乡交通一体化示范县和全省“四好农村路”示范县，新改建农村公路300公里、农村危桥15座、安保工程150公里。完成农村危房改造1260户。开工建设鹰潭220千伏月湖南、贵溪110千伏铜都等输变电工程，加快农村电网升级改造。加强地质灾害预防与治理。着力培育一批特色小镇，推动公共服务、基础设施、人才培养向农村延伸和倾斜。培养造就一支懂农业、爱农村、爱农民的“三农”工作队伍，支持和鼓励农民就业创业，持续提高农民收入水平。弘扬勤劳节俭、遵德守礼、孝老敬老的文明乡风民风。健全自治、法治、德治相结合的乡村治理体系，完善村民理事会制度，推进农村社区建设试点，让农村成为安居乐业的美丽家园。

（五）实施打造改革开放高地行动计划，着力激发“两都一城”建设新活力。坚持以改革增红利，以开放拓空间，以项目促发展，进一步增强发展动能，累积加快发展的后发优势。

突破重点领域、关键环节改革。纵深推进“放管服”改革，梳理企业群众办事“最多跑一次”甚至“一次不跑”事项清单，125项政务服务事项实现“一号申请、一窗受理、一网通办”，发放“惠民一卡通”84.3万张。适时启动新一轮党政机构改革，深化综合执法体制改革，深入推进余江县锦江镇经济发达镇行政管理体制改革，实现市县两级行政许可标准化“全覆盖”。稳妥推进事业单位分类改革。深化监察体制改革。深入推进“多证合一”改革、“双随机一公开”监管，全面开展中介服务“减时、降费、提质”专项行动。抓好国企发展混合所有制试点和职工持股试点工作，推进国有资本投资、运营公司试点，盘活整合国有资产。壮大产业引导基金和股权投资基金，大力发展普惠金融，支持各类资本发起设立各类法人金融机构，促进互联网金融、大数据金融、众筹金融等新业态发展，新增“新三板、四板”挂牌展示企业4家、35家。启动第三次全国土地调查。

掀起大干项目、干大项目热潮。加强项目谋划，初步安排大中型建设项目370个，年度计划投资458.5亿元，新开工建设123个、建成184个。加快项目建设，推进凤凰光学光电设备等一批在建项目投产运营，推动花桥水利枢纽工程、贵溪至资溪高速公路等一批重大项目取得实质进展，抓好通用机场、鹰潭至鄱阳城际铁路、鹰潭港等项目前期工作。强化要素保障，积极争取政策性、开发性、商业性金融机构支持，推进批而未用和闲置低效用地消化利用，畅通项目审批“绿色通道”，从严打击强揽工程、恶意阻工等违法行为。落实鼓励民间投资各项政策，推广政府和社会资本合作模式，引导社会资本进入物联网、市政建设、社会民生等领域。

推动全方位、宽领域开放。创新区域合作机制，全力对接昌鹰产业走廊建设，积极参与长江经济带、长江中游城市群、赣东北扩大开放合作等国家、省重大战略分工协作。实施招大引强攻坚计划和“央企入鹰、鹰商回归”提升工程，做到招商引资与招才引智并重、招引新企业与鼓励存量企业技改扩张并重、引进企业与孵化企业并重。全市引进省外2000万元以上项目资金379亿元、增长9%，实际利用外资3.11亿美元、增长8%。坚持“引进来”与“走出去”相结合，引导优势企业产能出海、境外投资，外贸出口增长3%左右。

（六）实施打好“三大攻坚战”行动计划，着力肩负“两都一城”建设新责任。坚持抓重点、补短板、强弱项，坚决打好防范化解重大风险、精准脱贫、污染防治三大攻坚战，不断夯实全面建成小康社会的基础。

坚决打好精准脱贫攻坚战。深入推进十大脱贫攻坚工程，全年完成脱贫5451人，退出12个贫困村。大力实施产业扶贫，推行“扶贫产业信贷通”，抓好特色种养业、乡村旅游、光伏产业、加工产业、农村电商、农业科技服务等扶贫“六项任务”，推进股份合作型、龙头带动型、资产收益型、托管寄养型、土地流转型、混合发展型“六种模式”，巩固提升扶贫产业全覆盖成效，实现建档立卡贫困户家家都有稳定增收产业。严格做好兜底保障脱贫，统筹推进健康、教育等扶贫再提升工程，全面落实农村贫困人口大病医疗商业补充保险政策，稳定实现农村贫困人口“两不愁、三保障”。深入开展结对帮扶，鼓励和引导社会力量参与扶贫。注重扶志与扶智相结合，激发贫困群众脱贫的内生动力，确保脱贫成效经得起实践和历史检验。

坚决打好污染防治攻坚战。深入实施“气十条”，启动新一轮大气污染防治行动计划，推进贵电5号、6号机组完成超低排放改造和有色冶炼企业达标排放，抓好工业园污染治理、施工工地及城市道路扬尘整治、农业秸秆综合利用和禁烧、机动车达标排放，打赢蓝天保卫战。深入实施“水十条”，继续推进“清河行动”，建立“湖长制”，持续开展江河湖水域问题专项整治，大力推进信江市区段流域生态综合治理，配合市人大完成信江饮用水水源环保地方立法工作，全市中小型水库、山塘全面退出承包合同。深入实施“土十条”，开展土壤污染状况详查和治理修复，完成农用地土壤详查，抓好土壤项目库建设和管理。推进农业面源污染整治，加大畜禽养殖场污染整治力度，规模畜禽养殖场配套建设废弃物处理设施比例达85%以上。加大环境执法监管力度，切实抓好中央和省环境保护督察组反馈问题整改，以“零容忍”态度坚决惩治环境违法行为。

坚决打好防范化解重大风险攻坚战。加强风险监测预警，妥善处理风险案件，坚决守住不发生系统性和区域性风险的底线。加强商品房市场监管，加大非住宅商品房去库存力度，加快发展住房租赁市场，建立多主体供应、多渠道保障、租购并举的住房制度。防范金融风险，建立风险识别和预警机制，打击非法集资、金融诈骗、各类高利贷活动，加强“影子银行”管控，规范民间金融发展。防范政府债务风险，规范举债行为，加快置换存量债务，推进政府融资平台市场化，确保债务余额只减不增。推进信用鹰潭建设，加强社会信用体系建设，加大对失信行为的惩戒力度。

（七）实施保障和改善民生行动计划，着力提升“两都一城”建设新福祉。坚持人人尽责、人人享有，在幼有所育、学有所教、劳有所得、病有所医、老有所养、住有所居、弱有所扶上不断取得新进展，使群众获得感幸福感安全感更加充实、更有保障、更可持续。

进一步完善社会保障。坚持就业优先战略和积极就业政策，深入推进贵溪、余江国家农民工返乡创业试点建设，统筹抓好高校毕业生、困难群众就业，大力培育创业孵化基地、创业特色小镇等载体，城镇新增就业1.96万人，新增转移农村劳动力1.25万人。扎实推进全民参保工作，全面实施机关事业单位养老保险制度改革和职业年金制度，稳步推进“五险统征”工作，力争城镇职工养老保险参保达24.2万人、城乡居民养老保险参保达44.5万人、基本医疗保险参保达118万人。提高城乡低保标准，实现农村低保标准与贫困标准融合。健全社会救助体系，建立社会关爱援助体系，强化农村留守儿童关爱保护。

进一步发展社会事业。围绕创建国家卫生城市，全面推进公立医院综合改革，大力实施全面提升县级公立医院综合能力三年行动计划，加快推进智慧医疗信息平台建设，不断改善群众就医体验。围绕打响“学在鹰潭”品牌，实施第三期学前教育三年行动计划，着力解决好婴幼儿照护和儿童早期教育服务问题。加快实施中小学生示范性综合实践基地、市二中南校区改扩建、市实验中学重建、高桥中学、新周塘中学、农村学校“改薄”等项目，着力解决中小学生课外负担重、“择校热”“大班额”等突出问题，余江县义务教育均衡发展通过国家督导验收。实施普及高中阶段教育攻坚计划，支持社会力量办学，推动高等教育内涵式发展和职业教育、网络教育特色化发展。围绕国家食品安全示范城市创建工作，建成日处理100吨餐厨垃圾处理厂，打造“从田间到餐桌”全产业链质量安全管控体系。实施“文化+”工程，促进文化与旅游、科技等深度融合，加快“四馆”等项目建设。完善全民健身基础设施，备战省第十五届运动会。推进老年大学建设，支持社会资本设立养老机构，打造50个居家养老服务中心。

进一步创新社会治理。加强社会治安综合治理创新，推行网格化管理、社会化服务和信息化支撑，夯实社会治理基础。推进法治鹰潭建设，健全覆盖城乡的法律服务体系，完善人民调解、行政调解、司法调解联动工作机制。强化社会治安防控体系建设，严厉打击各类违法犯罪活动，确保社会治安平稳有序。深化信访制度改革，压实信访工作责任，引导群众依法理性有序表达诉求。严格落实安全生产责任制，确保人民群众生命财产安全。强化意识形态工作，加强互联网管理，牢牢掌控网络意识形态主导权。深入推进军民融合发展，加强国防教育、人防民防、民兵预备役建设。与此同时，进一步做好统计、档案方志、民族宗教、计划生育、广播电视、涉台、外事侨务、机关事务、气象和残疾人等工作，支持各民主党派、工商联、知识分子联谊会和工会、共青团、妇联等人民团体的工作。

三、加强政府自身建设

深入贯彻习近平新时代中国特色社会主义思想，坚定不移推进全面从严治党，依法行政、从严治政、廉洁从政，努力建设人民满意信赖的政府。

政治坚定，不忘初心。不忘初心，方得始终。我们将牢固树立“四个意识”，坚决维护以习近平同志为核心的党中央权威和集中统一领导，坚定执行党的政治路线，严格遵守政治纪律和政治规矩，始终在政治立场、政治方向、政治原则、政治道路上同党中央保持高度一致。始终坚持以人民为中心的发展思想，把人民群众所想所盼作为政府工作的第一追求，用真心真情践诺初心、温暖人心、赢得民心，更好地满足人民日益增长的美好生活需要。

敢于担当，不辱使命。有多大担当才能干多大事业，尽多大责任才会有多大成就。我们将不断增强改革创新、科学发展、依法执政、群众工作、狠抓落实、驾驭风险等能力和水平，推动质量变革、效率变革、动力变革，更好地适应新时代发展要求。牢牢把握发展第一、担当为重、实干托底的工作定位，在加快发展上担当重任，在改善民生上担负重托，在破解难题上担起重担，少说不能为、多想怎么办，少求面上光、多务民生实，少图眼前利、多谋长远计，用担担子、扣扣子、钉钉子的精神，把定下的事干实干好，把承诺的事办成办妥。

依法行政，不越边界。有权不能任性，用权更不能任性。我们将始终坚持法定职责必须为、法无授权不可为，加快建设法治政府，做到授予有据、行使有规、施政有信、监督有效，既让权力“进笼子”，又让权力“晒太阳”，确保行政权力始终在法治轨道上运行。自觉接受人大法律监督、政协民主监督、社会监督、司法监督，认真听取各民主党派、工商联、无党派人士、各人民团体的意见建议，严格执行民主集中制，紧紧依靠集体智慧推动工作，努力把政府系统建设成一个战斗的团队、奔跑的团队、温暖的团队。

廉洁从政，不碰底线。贪欲是最真实的贫穷，满足是最真实的财富。我们将始终把廉洁从政作为政府工作的底线，扎实开展“不忘初心、牢记使命”主题教育，落实主体责任，严肃党内政治生活，推进“两学一做”学习教育常态化制度化。按照“纠正‘四风’不能止步，作风建设永远在路上”的要求，严格执行中央八项规定精神，从严监管公共资金、公共资源、公职人员，切实做到严守纪律不逾矩，严以用权不任性，严以律己不谋私，维护好鹰潭“山清水秀”的政治生态，让想干事、能干事、干成事成为全市上下的新常态。

各位代表，走进新时代，展望新征程，我们深感使命光荣，责任重大。让我们更加紧密地团结在以习近平同志为核心的党中央周围，在市委的坚强领导下，不忘初心，牢记使命，努力开创决胜全面小康、建设富裕美丽幸福鹰潭新局面，奋力谱写新时代中国特色社会主义发展的鹰潭篇章！
